# Immune checkpoint inhibitor-induced diabetes can potentially be effectively treated with infliximab: a case report of two patients

**DOI:** 10.3389/fendo.2025.1697724

**Published:** 2025-11-07

**Authors:** Noam Savion Gaiger, Michael E. Hurwitz, Navid Hafez, Harriet M. Kluger, Kevan C. Herold, Ana Luisa Perdigoto

**Affiliations:** 1 Medical Oncology, Yale School of Medicine, New Haven, CT, United States; 2 Hematology Oncology, Cedars Sinai, Los Angeles, CA, United States; 3 Department of Internal Medicine, Yale School of Medicine, New Haven, CT, United States; 4 Department of Immunobiology, Yale School of Medicine, New Haven, CT, United States; 5 Department of Internal Medicine, Veterans Administration Hospital, West Haven, CT, United States

**Keywords:** immune checkpoint inhibitors, diabetes mellitus, immune related adverse events, TNF-α, infliximab

## Abstract

Immune checkpoint inhibitor-induced diabetes is a potentially severe and life-threatening complication of immune checkpoint inhibitor therapy in patients with advanced malignancies. It is typically an irreversible complication due to the complete destruction of pancreatic beta cells that requires ongoing insulin treatment, and patients often exhibit labile diabetes. Inflammatory cytokines, including TNF-α, are thought to play a role in the development of this form of diabetes, as they do in spontaneous autoimmune diabetes. TNF-α has also been implicated in the development of other immune-related adverse events caused by immunotherapy, and infliximab, a TNF-α monoclonal antibody, has shown efficacy in several of these complications. We tested whether infliximab could preserve beta-cell function in immune checkpoint inhibitor-induced diabetes. We present two cases in which infliximab treatment appeared to halt beta-cell destruction, as evidenced by maintenance of C-peptide levels, as well as improved clinical outcomes in terms of diabetes control.

## Introduction

Immune checkpoint inhibitors (ICIs) have transformed the landscape of cancer treatment, demonstrating efficacy and improved survival for patients with various advanced cancers. However, administration of these agents results in immune-related adverse events (irAEs), affecting various organs, including pancreatic islets. Destruction of beta cells (β cells) in the pancreas results in diabetes clinically akin to type 1 diabetes (T1D) (1).

ICI-induced diabetes (ICI-DM), primarily observed with anti-PD-1 or anti-PD-L1 therapies, affects approximately 0.2%–1.9% of patients treated with ICIs, but some series with more aggressive ICI regimens have reported up to 11% developing hyperglycemia (1, 2). ICI-DM presents acutely with severe hyperglycemia, often with diabetic ketoacidosis (DKA), which is potentially life-threatening. Patients have low or undetectable C-peptide consistent with rapid β-cell destruction (3, 4). The precise mechanism, though still not well understood, is attributed to autoimmune destruction of β cells, triggered by ICI-mediated T-cell activation (5). Treating ICI-DM involves promptly starting insulin therapy, and subsequent management resembles that of T1D (1). In our experience, corticosteroids are not effective in reversing ICI-DM and pose the risk of further exacerbation of hyperglycemia.

Specific immunosuppressive therapies targeting the pathways causing irAEs including ICI-DM are not yet established. Our previous studies, in NOD mice, suggested that inflammatory cytokines were required to induce ICI-DM (5). TNF-α has been found to have a role in spontaneous T1D since two clinical trials with anti-TNF-α agents (etanercept and golimumab) attenuated loss of C-peptide in patients with new-onset disease (6, 7). Infliximab, a TNF-α monoclonal antibody (mAb), is frequently used in the treatment of TNF-α-driven autoimmune diseases such as rheumatoid arthritis and inflammatory bowel disease (8). Anti-TNF-α can also successfully treat various irAEs, particularly colitis (8, 9). For colitis, there is evidence that infliximab may be safer than corticosteroids and lead to faster symptom resolution (9).

A case study described the successful treatment of ICI-DM in a patient with melanoma following treatment with infliximab administered for co-occurring arthritis (10). Treatment resulted in the reversal of β-cell dysfunction, return of insulin sensitivity, elimination of the need for insulin treatment, and normalization of HbA1c levels. However, the patient had also been treated with steroids recently and was not insulinopenic, raising the question of whether infliximab could be efficacious in other cases of ICI-DM. Based on these background data and clinical experience, we tested whether infliximab could mitigate β-cell loss in patients with ICI-DM.

Here, we present two cases of patients with ICI-DM successfully treated with infliximab, providing additional evidence that in patients with ICI-DM with some residual endogenous β-cell function, infliximab may halt the progression of β-cell loss.

## Case reports

### Case 1

A patient approximately 60 years of age with melanoma, with metastases to the liver, lung, and peritoneum/omentum, was treated with 3 mg/kg of ipilimumab (anti-CTLA-4) and 1 mg/kg of nivolumab (anti-PD-1) followed by nivolumab monotherapy. Following the first cycle, the patient experienced fevers that resolved spontaneously, and after the second cycle, the patient developed diarrhea that improved with corticosteroids but completely resolved with infliximab. At that point, the patient had a deep tumor response to therapy and continued with nivolumab monotherapy. Following the ninth cycle, the patient developed mild uveitis, which was effectively treated with corticosteroid eyedrops. After the 21st cycle, the patient still had a deep response and treatment was stopped. Two months after the last cycle, 826 days after starting ICIs, the patient presented with polydipsia, polyuria, and fatigue and was found to have a blood glucose level of 327 mg/dL. Notably, the patient had not received any steroids prior. Other laboratory evaluation revealed a normal beta-hydroxybutyrate level at 0.09 mmol/L (normal <0.27 mmol/L), and the bicarbonate level was in the normal range. A random C-peptide level was 2.6 ng/mL (normal range 1.1–4.4 ng/mL). The HbA1c, which was in the normal range prior, was 8.8%. GAD65, IA-2, and insulin autoantibodies were negative. An insulin basal/bolus regimen was initiated.

**Figure 1:**
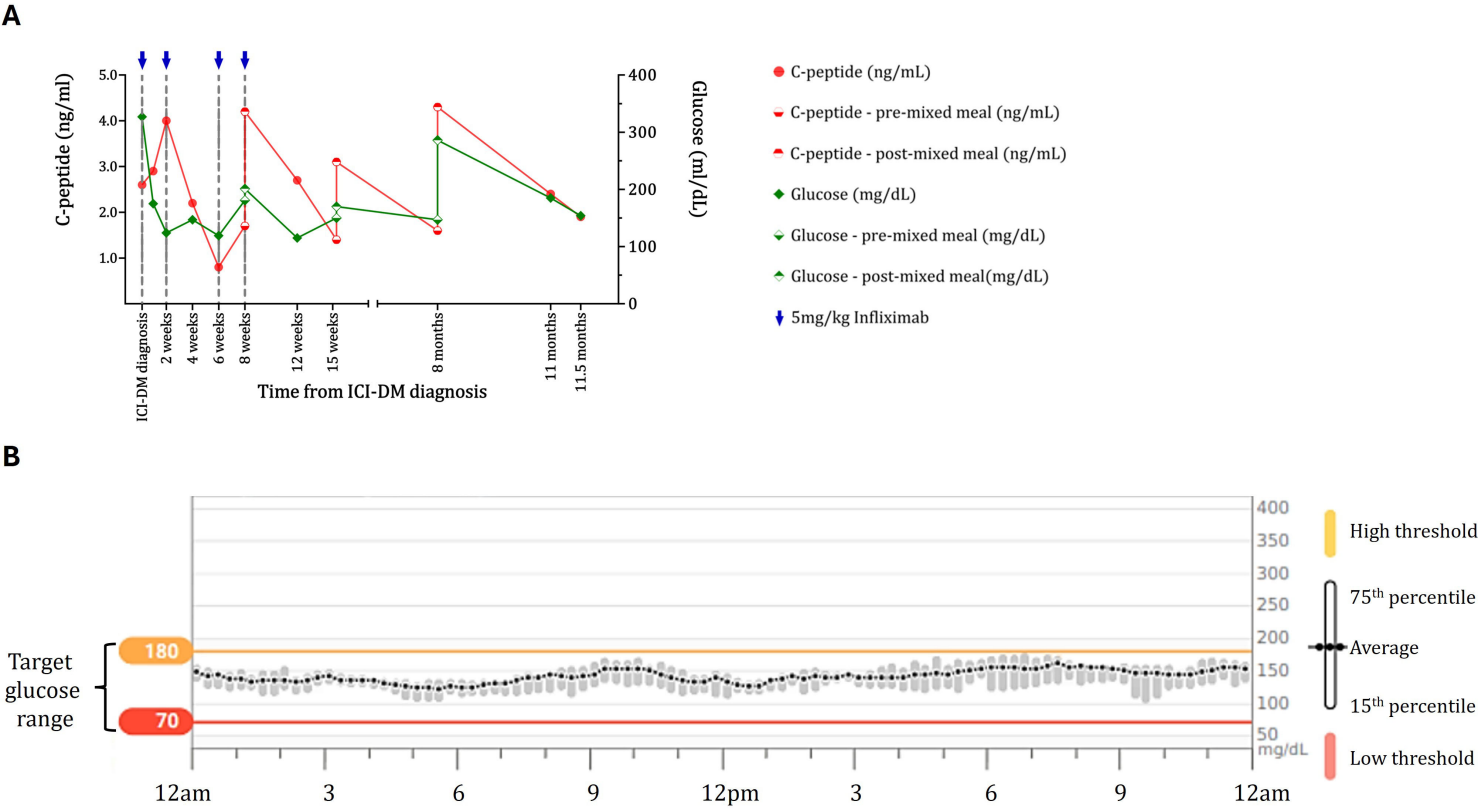
Maintenance of beta-cell function and diabetes control in the case 1 ICI-DM patient treated with infliximab. **(A)** C-peptide (red) and blood glucose (green) data for the case 1 patient show maintenance of C-peptide on both random checks and response to a mixed meal. For the response to the mixed meal, baseline glucose and C-peptide levels (diamond and circle filled on the bottom, respectively) 2 h after drinking Ensure Plus (diamond and circle filled on the top) are shown. Blue arrows indicate doses of infliximab. C-peptide levels and responses are maintained for almost a year after diagnosis and initiation of infliximab. **(B)** Continuous glucose monitoring data for the case 1 patient 168 days following diabetes diagnosis. Blood glucose data for a 2-week period showing average blood glucose (with 15th and 75th percentiles) over 24 h while the patient was on glargine 10 units and lispro 3 units before meals. The patient had excellent control (93% of blood glucose levels are in the target range) without significant blood glucose fluctuations or lows typically observed in patients with ICI-DM. Yellow line = high threshold (180 mg/dL); red line = low threshold (70 mg/dL).

Due to the clinical evidence of endogenous β-cell reserve, the patient was treated with four doses of infliximab, 5 mg/kg IV. Following infliximab infusion, C-peptide levels improved and stabilized (**Figure 1A**). Liver function tests remained in the normal range. Response to a mixed meal was assessed on three occasions, and following infliximab, C-peptide levels increased more than twofold within 2 h following the consumption of 47 g of carbohydrate (**Figure 1A**). Following infliximab treatment, diabetes control was excellent with 93% time in range, 6% time above range, and 1% time below range (**Figure 1B**). Based on glycemic control, short-acting insulin at meals was tapered off, and treatment was transitioned to metformin with continuation of a low dose of glargine due to concern regarding the adverse effects of other antihyperglycemic agents. The HbA1c remained at 7% more than 349 days after diabetes onset. The infliximab treatment did not appear to interfere with clinical response to ICI. The tumor remains in deep response, with resolution of >80% of the tumor bulk.

### Case 2

A patient approximately 70 years of age with metastatic prostate cancer to the bone and lymph nodes had been previously treated with multiple lines of therapy (bicalutamide, leuprolide, docetaxel, and enzalutamide) with disease progression. The patient was started on immunotherapy in a clinical trial involving 300 mg of PF-06801591 (anti-PD-1) and 80 mg of tremelimumab (anti-CTLA-4) as well as a viral vaccine encoding prostate cancer-associated antigens. A month following the third cycle, 259 days after starting immunotherapy, treatment was held per protocol for lipase elevation to 125 U/L (normal 11–55 U/L). There was no clinical or radiographic evidence of pancreatitis.

**Figure 2:**
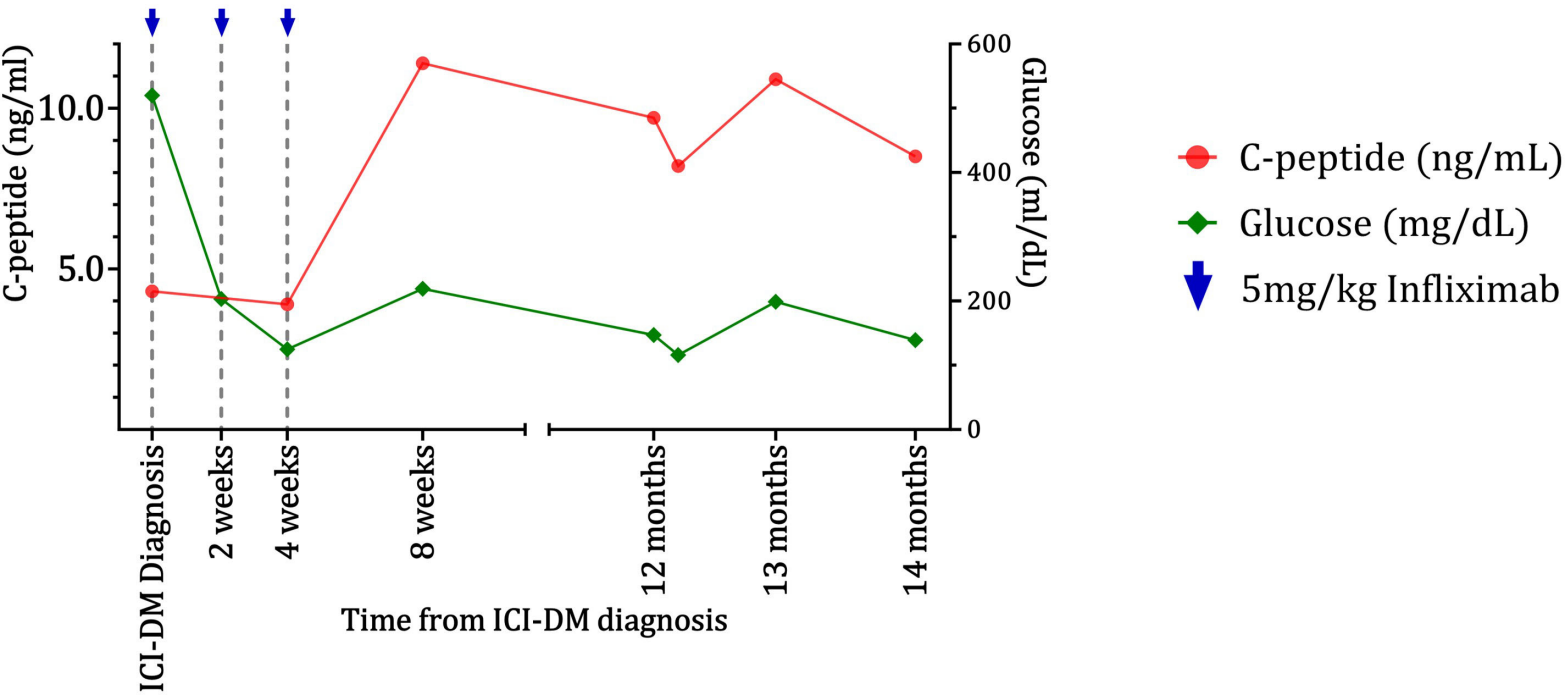
Improvement and maintenance of beta-cell function as indicated by C-peptide levels for the case 2 patient following infliximab treatment. C-peptide (red) and glucose (green) data for the case 2 patient show maintenance of C-peptide for over 1 year after worsening of diabetes and infliximab injections.

The patient had a 7-year history of well-controlled T2D with HbA1c prior to immunotherapy initiation (7.2% on diet alone). Worsening hyperglycemia (glucose = 520 mg/dL) prompted hospital admission 14 days after the lipase elevation and 273 days after initiation of immunotherapy. Beta-hydroxybutyrate was slightly elevated at 0.83 mmol/L (normal <0.27 mmol/L), but the bicarbonate level was 21 mmol/L (normal range 20–30 mmol/L). A random C-peptide was 4.3 ng/mL. GAD65, IA-2, and insulin autoantibodies were negative. The HbA1c had worsened to 9.2%. The patient had not received steroids. The patient was started on a basal/bolus insulin regimen and was treated with 5 mg/kg of infliximab approximately every 2 weeks and received three doses. A mild elevation in liver function tests was observed but subsequently resolved. C-peptide levels improved for more than a year after infliximab treatment (**Figure 2**). Glucose control enabled the discontinuation of insulin treatment 58 days after it was started and switched to metformin only. The prostate cancer was subsequently treated with docetaxel followed by cabazitaxel and radium-223. Despite requiring prednisone with chemotherapy regimens, the patient did not require additional insulin, and HbA1c was 5.8% 650 days after the hospital admission for hyperglycemia. The patient remained on metformin only until death from prostate cancer 749 days after presenting with hyperglycemia.

## Discussion

ICI-DM is typically irreversible and potentially life-threatening as a result of DKA due to β-cell loss. Our preclinical studies have shown that there is production of inflammatory cytokines that have direct toxic effects on β cells and activate other immune cells (5). Previous findings further support the role of TNF-α in cytokine-induced β-cell destruction through mediating lymphocytic infiltration and promoting local islet inflammation (6). A subset of patients present prior to total β-cell loss, when residual function may still be preserved through modulation of cytokine-induced inflammation, such as TNF-α blockade.

We present two cases of ICI-DM in which early administration of infliximab appears to have halted the complete loss of β-cell function, as evidenced by C-peptide levels. The typical course in patients with ICI-DM is a rapid decline in C-peptide levels, with very few patients retaining detectable function more than 100 days after the development of diabetes (3). One study found that C-peptide was low in 91.6% of patients at presentation and 100% of patients on follow-up (4). These two cases demonstrate maintenance of C-peptide significantly longer than usually seen with ICI-DM after treatment with infliximab.

Our first patient did not have a prior history of diabetes, while the second had mild disease treated with diet only. T2D is common and some patients with ICI-DM present as worsening of stable disease as in this patient. There are no clear diagnostic criteria to distinguish this form of diabetes from others including spontaneous autoimmune diabetes. Our assessment was based on the precipitous deterioration of the patient’s clinical status without complicating treatments such as glucocorticoids. It is promising that the patient’s endogenous β-cell function was preserved following infliximab for as long as it did, allowing the patient to discontinue insulin, potentially due to modulation of cytokine-induced inflammation and β-cell damage.

Maintenance of C-peptide in patients with T1D has been associated with better glycemic control, reduced risk of hypoglycemia, and reduced complications (11). From our clinical experience, ICI-DM patients with loss of C-peptide demonstrate labile glucose control including frequent hypoglycemia. Case 1 suggests that C-peptide maintenance through infliximab results in improved control without significant hypoglycemia while on insulin. Furthermore, we were able to discontinue short-acting insulin, which has not been possible with other ICI-DM patients. The second patient passed away, and a mixed meal response test was not performed; however, C-peptide levels were maintained 1 year after ICI-DM diagnosis and infliximab therapy, suggesting retained β-cell function.

As no formal guidelines exist for infliximab use in ICI-induced irAEs, dosing was based on consensus recommendations for steroid-resistant immune-related colitis, balancing clinical benefit against potential interference with antitumor treatment (12). We followed C-peptide levels to ensure ongoing maintenance of C-peptide and also considered cancer response status. The first patient, who had an excellent oncologic response, received four doses; additional doses were considered given the continued maintenance of C-peptide levels, but treatment was discontinued after a local allergic reaction to infliximab. Infliximab therapy was unlikely to have affected the response of these patients’ tumors to ICIs. Both preclinical and clinical data suggest that antitumor response is maintained in the setting of short-term TNF inhibition (8, 9). The biological half-life of the drug is 7–12 days. Investigations in patients who receive infliximab for rheumatic diseases indicate that its effects do not persist after mAb clearance. Patient 1 continued to have a good clinical response after treatment, and patient 2 had progression of disease prior to and following infliximab, prompting initiation of chemotherapy. We are limited in our ability to assess the possible impact of infliximab on tumor response given that we only have two patients, each with a different tumor treatment outcome. However, in both cases, infliximab did not seem to alter the response.

Further research is essential to assess the effectiveness and safety of TNF-α inhibitors in ICI-DM and to understand the effects of anti-TNF-α on the modulation of β-cell loss and antitumor immunity. These two cases suggest that in ICI-DM patients with residual β-cell function, as evidenced by preserved C-peptide levels, prompt administration of infliximab may result in recovery and/or maintenance of β-cell function with significant clinical implications for diabetes control.

## Data Availability

The original contributions presented in the study are included in the article/supplementary material. Further inquiries can be directed to the corresponding author.
